# Meta-analysis suggests evidence of novel stress-related pathway components in Orsay virus - *Caenorhabditis elegans* viral model

**DOI:** 10.1038/s41598-019-40762-9

**Published:** 2019-03-13

**Authors:** Priyanka Mishra, Jessica Ngo, Jahanshah Ashkani, Frederic Pio

**Affiliations:** Molecular Biology and Biochemistry Department, Simon Fraser University, Burnaby-V5A1S6, British Columbia, Canada

## Abstract

The genetic model organism, *Caenorhabditis elegans* (*C*. *elegans*), shares many genes with humans and is the best-annotated of the eukaryotic genome. Therefore, the identification of new genes and pathways is unlikely. Nevertheless, host-pathogen interaction studies from viruses, recently discovered in the environment, has created new opportunity to discover these pathways. For example, the exogenous RNAi response in *C*. *elegans* by the Orsay virus as seen in plants and other eukaryotes is not systemic and transgenerational, suggesting different RNAi pathways between these organisms. Using a bioinformatics meta-analysis approach, we show that the top 17 genes differentially-expressed during *C. elegans* infection by Orsay virus are functionally uncharacterized genes. Furthermore, functional annotation using similarity search and comparative modeling, was able to predict folds correctly, but could not assign easily function to the majority. However, we could identify gene expression studies that showed a similar pattern of gene expression related to toxicity, stress and immune response. Those results were strengthened using protein-protein interaction network analysis. This study shows that novel molecular pathway components, of viral innate immune response, can be identified and provides models that can be further used as a framework for experimental studies. Whether these features are reminiscent of an ancient mechanism evolutionarily conserved, or part of a novel pathway, remain to be established. These results reaffirm the tremendous value of this approach to broaden our understanding of viral immunity in *C*. *elegans*.

## Introduction

Despite being of intermediate complexity when compared to single-celled eukaryotes and mammals, *Caenorhabditis elegans* (*C*. *elegans*) offers an ideal system for genome organization and functional studies with a gene complement that is remarkably conserved in vertebrates (38% with human)^1^. The genome of *C*. *elegans* contains as many genes as humans and many of them are functional homologues, which allows for direct functional comparisons between the two organisms. Therefore, *C*. *elegans* has been considered as a model for investigating innate immunity, particularly in organismal stress-resistance and longevity^2^. In addition, many genetic strains of *C*. *elegans* and RNA interference (RNAi) machinery allows loss of function phenotype studies for almost each gene. These powerful tools, as well as other state-of-the-art reverse genetic technologies, have made *C*. *elegans* an organism with one of the best functionally annotated genome^3,4^. The use of this model to study neuroscience^5^ and host-pathogens interaction mechanisms has given great insight. However, a surprisingly large percentage of its gene repertoire is still without known function, particularly its interactions with microbes^6–8^. Since many pathogens were discovered in wild *C*. *elegans* strains the opportunity to study their interaction with the host was not available until recently. This raises the possibility that many genes of unknown function may be dysregulated, once the pathogen is reintroduced into a genetics laboratory strains such as Bristol N2. The prospect for unravelling novel pathway components activated specifically by these pathogens is increased.

*C*. *elegans* feeds on diverse microbial flora, including bacterial and fungal pathogens, from which the ecology and host/pathogen interactions remains poorly understood. As a result, several studies provided new insights into how *C*. *elegans* responds to bacterial or fungal infections by activating important pathways such as Mitogen-activated protein (MAP) kinase, transforming growth factor beta (TGF-β) and insulin signaling^9^. In this regard, the discovery of the Orsay virus, a natural intracellular pathogen of *C*. *elegans*, opened new possibilities to study multiple facets of host-virus interactions using the nematode as a model organism^10^. After viral infection by Orsay virus in *C*. *elegans*, the RNAi and ubiquitin pathways act as a protection mechanism against viral infection^11,12^ and more insights will probably be discovered considering that a significant fraction of the *C*. *elegans* genes that are dysregulated by Orsay virus infection are currently unannotated^9,12^. Annotating them may reveal novel pathway components that have been missed due to the lack of data mentioning their functional importance.

Since its discovery in *C*. *elegans*, RNAi has proven to be essential during development and in disease. Exogenous RNAi spreads throughout the organism between cells and can be passed between generations; however, there have been disagreements pertaining to the possible endogenous role of the RNAi pathway. By spreading within the infected organism and between generations, the endogenous role of RNAi pathway would be advantageous against viral infection in plants as antiviral RNAi is systemic and the spread of RNAi between cells provide protection against subsequent viral infection^13^. However, recent studies, on viral infected *C*. *elegans* by *Nodavirus Orsay*, have found that in contrast to the exogenous RNAi pathway, the antiviral RNAi pathway targeted against this virus does not spread systemically throughout the organism and is not deliverable between generations^11,13^.

In the context of viral infection, by considering the involvement of different RNAi pathways in *C*. *elegans* and in plants as well as some evidence suggesting that novel pathway component may exist, we aimed at characterizing these differences through the assessment of gene expression data from publicly available databases. We have applied a meta-analysis approach and focused on trying to annotate a function to the unknown genes that are being dysregulated when *C*. *elegans* is being challenged by Orsay virus. Furthermore, the recent discovery of a novel Nodavirus Endogenous Viral Element (EVE) in the genome of *Bursaphelenchus xylophilus*, a plant parasitic nematode, significantly highlight the potential of comparing and analyzing gene expression profile from different organism^14^. These novel features of viral innate immune response may be an ancient mechanism evolutionarily conserved and as a result relevant to human.

## Results and Discussion

To identify the differentially expressed genes when *C*. *elegans* is infected by Orsay virus, the GEO (GSE41056) dataset was analyzed by GEO2R https://www.ncbi.nlm.nih.gov/geo/geo2r/). Through differential gene expression analysis between non-infected versus infected samples (n = 4) we obtained a ranked list of 250 differentially expressed genes with a *p-*values below the significant threshold (*p-value* < 10^−3^). The Gene Ontology (GO) annotation of the differentially-expressed genes, by Gene Set Enrichment Analysis (GSEA) in PANTHER^15^ was carried out. Our results revealed that most of the differentially-expressed genes were involved in DNA repair, stress, catabolism, catalytic activity and nucleic acid binding (Table 1).

**Table 1:** Enrichment factors of the GO categories (^#^Number of genes that were annotated in each category) determined by GSEA for the 250 genes differentially expressed after Orsay virus infection.

Panther GO	^#^	Expected	Fold Enrichment	^±^*p*-value (0.05 cutoff)
DNA repair	7	1.09	6.40	+2.58E-02
Stress	17	3.52	4.83	+2.48E-05
Catabolism	13	2.74	4.74	+9.44E-04
Cell cycle	24	5.89	4.07	+1.45E-06
Catalytic activity	15	3.86	3.88	+1.90E-03
Regulation	15	3.88	3.86	+2.02E-03
Translation	13	3.45	3.77	+1.00E-02
Nitrogen compound metabolism	20	5.61	3.56	+2.23E-04
Phosphorylation	14	4.41	3.18	+3.13E-02

The *p*-values were adjusted by Bonferroni correction for multiple testing.

However, among the 250 differentially-expressed genes, we observed a marked over representation of genes with unknown function, which were annotated as unclassified in our GSEA. Remarkably, among these genes, 17 were identified as top differentially-expressed genes with *p*-value ≤ 0.0001 (Table 2). Below this *p*-value, a mixture of annotated and unannotated genes were present. Careful manual examination revealed that the level of differential-gene expression between infected versus non-infected samples was below two fold. For example, the gene *tbc-9* listed just after *sdz-6*, which is the last gene taken from the list of 17 gene data sets, has an expression value of 7.2 for the non-infected versus 7.8 for the infected sample. This is less than a two-fold difference (1.1 fold). As a result we did not consider adding more genes for analysis in this study.

**Table 2:** The 17 top ranking genes differentially-expressed based on the lowest *p*-value that have no known function.

Gene symbol	*p*-value
B0507.8	9.82E-11
F26F2.4	1.26E-09
F26F2.5	3.57E-09
B0507.10	4.69E-09
CELE_T26F2.3	7.51E-09
CELE_C43D7.4	2.01E-08
CELE_C17H1.6	4.54E-08
CELE_C17H1.7	6.22E-08
CELE_Y75B8A.39	1.81E-07
F26F2.2	3.79E-07
CELE_C43D7.7	4.07E-07
F26F2.3	4.08E-07
F26F2.1	4.81E-07
C49C8.2	1.73E-06
CELE_B0284.4	1.90E-06
F42C5.3	7.59E-06
sdz-6	1.21E-05

To investigate the association of the 17 uncharacterized genes to mechanisms specific to the RNAi pathway in *C*. *elegans*, protein BLAST^16^ analyses against non-redundant (nr) and plant databases were performed. The absence of significant hits (Expected threshold, E < 10) in the specified databases may indicate that these 17 uncharacterized genes are involved in a novel antiviral response. To further annotate their function, we performed a comparative modeling analysis using the three most commonly used methods PHYRE2^17^, SWISS-MODEL^18^ and IntFOLD3^19^ (Supplementary Figs S1, S2 and S3). Folding similarities between modeled and known structures can provide functional insight to the modeled sequence. The uncharacterized proteins were aligned to selected sequences of known structures scanned in the databases. The three-dimensional structures of the uncharacterized proteins were built using a chosen template based on the best statistical confidence scores. This is method specific but estimates and assesses the quality of the modeled structures. Finally, a functional inference on the uncharacterized *C*. *elegans* proteins was determined based on existing knowledge about the function of the known structures from which the models were built. For comparison purposes, since three methods were employed, we used the root mean standard deviation (RMSD) and percentage coverage to estimate the quality and the structural similarity of the model compared to the structural template. Table 3 summarizes the results. They indicate a 28% coverage on average for PHYRE2, 35% for SWISS-MODEL but a very high 89% for IntFOLD3 between the uncharacterized *C*. *elegans* protein sequences and known templates. This suggests that IntFOLD3 performed the best. While the coverage and RMSD vary between methods, in most instances there was a good consensus between the methods for the fold predicted using different templates (Supplementary Figs S1, S2 and S3). As such, sixteen structures were predicted to have the protein-α fold while two other sequences, CELE_C43D7.4 and F26F2.1 did not show consensus fold across methods. As a result no folds were assigned to them.

**Table 3:** Structural prediction and annotation using PHYRE2, SWISS-MODEL and IntFOLD3 of the top 17 unknown and highly differentially-expressed genes.

Gene symbol	PHYRE-2 RMSD (Å) (% Coverage)	SWISS-MODEL RMSD (Å) (% Coverage)	INTFOLD3 RMSD (Å) (% Coverage)	Annotation based on the template function
B0507.8	0 (26)	1 (25)	1 (98)	Transportin 3 – Protein import to nucleus (α -helix)
F26F2.4 isoform a	1 (66)	1 (32)	1 (82)	Synaptonemal complex 1 (α-helix)
F26F2.4 isoform b	1 (55)	**N**.**T**.	5 (90)	Similar to F26F2.4 isoform a
F26F2.5	1 (14)	1 (32)	4 (96)	Zinc finger domain (α-helix)
B0507.10	0 (26)	0 (32)	1(80)	Spectrin (α-helix bundle)
**CELE_T26F2**.**3**	**1 (99)**	**1 (96)**	**1 (97)**	**Dom3z protein Exoribonuclease (α/β- protein)**
CELE_C43D7.4	1 (21)	0 (25)	1 (90)	**No consensus fold across methods**
CELE_C17H1.6	1 (36)	0 (31)	1 (87)	Reticulocyte binding protein 5 signaling (α-helix bundle)
CELE_C17H1.7	0 (26)	1 (73)	1 (55)	Helical repeat domain (α-helix)
CELE_Y75B8A.39	0 (22)	0 (23)	1 (100)	De novo three helix bundle
F26F2.2	0 (17)	1 (46)	5 (87)	Adhesin coiled-coil (α-helix)
CELE_C43D7.7	0 (17)	0 (59)	1 (93)	Virb DNA binding domain (Helix-Turn-Helix)
F26F2.3	0 (18)	0 (15)	1 (95)	alpha/alpha toroid
F26F2.1	0 (1)	0 (6)	N.T. (0)	**No consensus fold across methods**
C49C8.2	0 (5)	0 (35)	1 (90)	Apolipophorin-iii lipid transport (5 helix bundle)
CELE_B0284.4	0 (31)	1 (28)	1 (90)	Ezra cytoplasmic domain (3 helix bundle repeat)
F42C5.3 isoform a	0 (35)	0 (17)	1 (91)	Reticulocyte binding protein 5 signaling (α-helix bundle)
F42C5.3 isoform b	0 (4)	0 (22)	1 (86)	Hof1p f-bar domain protein binding (coiled-coil)
sdz-6	0 (14)	0 (26)	1 (82)	Ectodomain of protein eff-1 (α-helix)

The root mean squared deviation (RMSD) between the best template and model were computed as well as the percent coverage (In brackets) of the *C*. *elegans* protein sequence by the model. (N.T.: No Template found).

Considering the RMSD for each superimposition, all the values for IntFOLD3 which gave the highest coverage, are generally below the threshold of 2 Å that represents a medium-resolution model. The two genes, F26F2.4 isoform b and F26F2.5 have a RMSD of 5 and 4 but with a very high coverage 90% and 96% respectively. Further visualization of the structural superimposition in PyMOL (template/model) for both genes revealed that their respective models were of good quality by looking at the overall fold conservation (The PyMOL Molecular Graphics System, Version 2.0 Schrödinger, LLC). It lead us to conclude that the prediction was valid (Supplementary Figs S1–S6).

For CELE_T26F2.3, an interesting result was found since the three methods could model this sequence into a unique 3D-structure in all attempts, even when employing different templates (PDB: 5bto) by the SWISS-MODEL server (Supplementary Data S1 and Figs S4–S6). Further investigation into the function of CELE_T26F2.3 using WormBase^20^ revealed that this gene has been annotated as a vertebrate homologue of a de-capping exonuclease called DXO/Dom3Z which is in line with the function of the template used in both PHYRE2 (Supplementary Fig. S1) and IntFOLD3 (Supplementary Fig. S3) for its modeling.

We thus propose that the folding of the majority of the 17 uncharacterized proteins investigated in this study have been predicted successfully and are in agreement across methods. The result of IntFOLD3 that shows very high coverage gives us a pool of structures that represent accurate folds (Supplementary Fig. S3). It is difficult at this point to use these models and their template to infer function for these sequences, since many of them are of bacterial origin. But it represents a step forward towards this direction since we present new information regarding the structure and function of these proteins that should be of interest to experimentalists for further functional studies. As a proof of concept, we have published a study that used comparative modeling and experimental design to gain further functional insights into a protein of unknown structure and function^21^.

**Figure 1:**
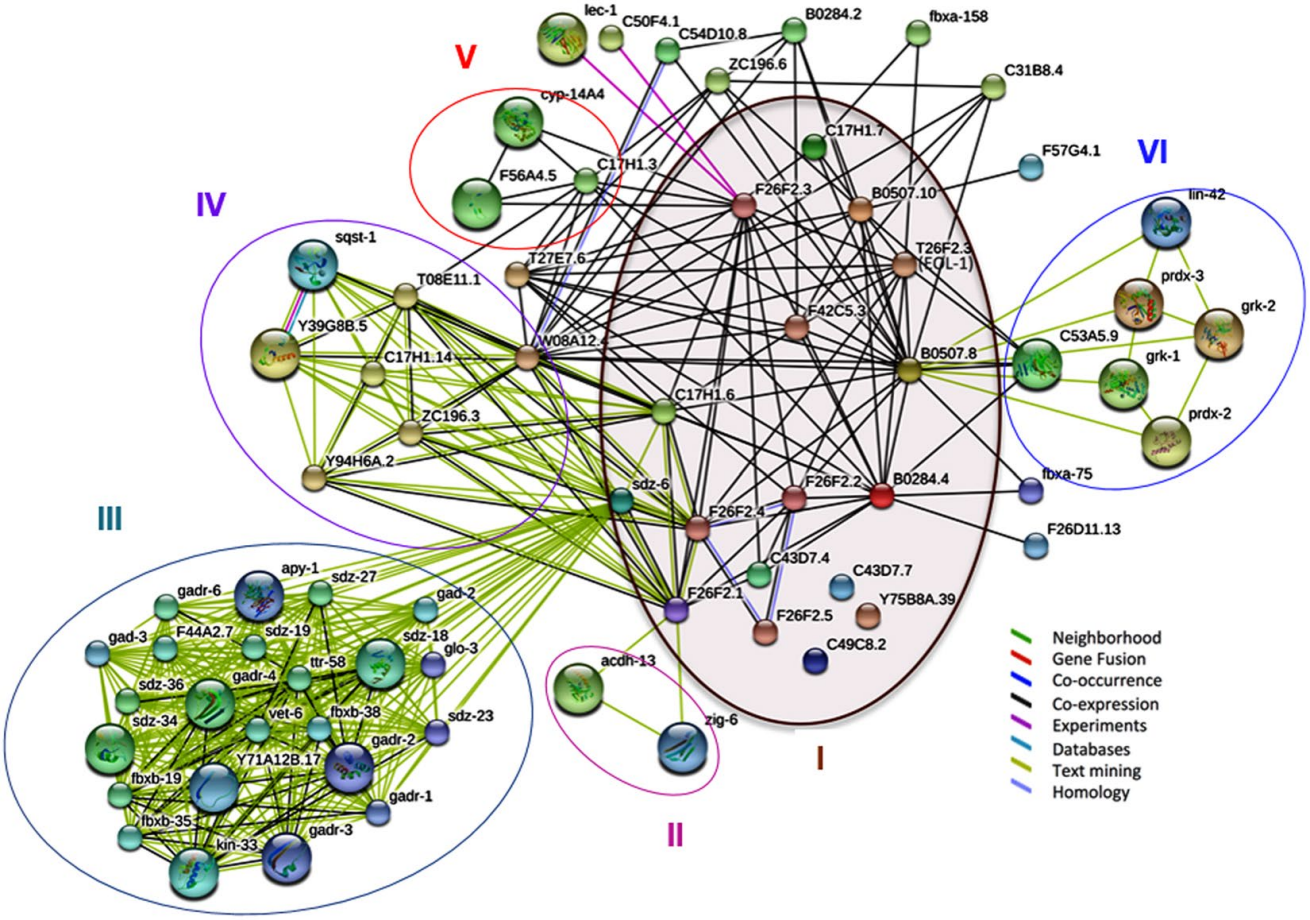
Protein-Protein interaction Network of the linkage between the 17 uncharacterized genes and their associated partners in STRING database (https://string-db.org). The circle represents groups of genes or clusters identified by text-mining and/or co-expression in one single paper.

In addition, we have further investigated the interacting partners or neighbors of the uncharacterized genes to shed light on their possible function. Figure 1 illustrates the protein-protein association network, with the 17 uncharacterized genes grouped into VI clusters. Each cluster represents a unique transcriptomics study where co-expression is highlighted by links in black, and when there is a co-occurrence of two genes (nodes) in the same published study, the interaction is visualized by a green link between them. The group of genes in the center (cluster I) represent the 17 genes from this study that are co-expressed but have no known function.

Further assessment of the link connecting the 17 uncharacterized genes showed 5 clusters representing different unique studies. F26F2.1 is connected to cluster II (*acdh-13* and *zig-6*) by text mining from a study identifying novel genes that extend the lifespan in *C*. *elegans* through insulin signaling, stress response and dietary restriction^22^. In this study, the lack of F26F2.1 expression in a knock down experiment by RNAi was shown to extend lifespan in the N2 strain. This result is in agreement with the study of Felix *et al*.^11^ as well as some unpublished results from our lab indicating that Orsay virus infection in *C*. *elegans* shortens the lifespan. *sdz-6* interact with cluster III genes. *sdz-6* has been annotated in WormBase as a gene involved in gastrulation^23^. This annotation came from the fact that *sdz-6* is co-cited with many co-expressed genes involved in gastrulation as reported in the unique study of Sawyer *et al*.^23^. In addition, *sdz-6* as well as F26F2.1, F26F2.4 and C17H1.6 are connected by co-expression and text mining to cluster IV that represent a study of Bakowski *et al*.^12^. In this latest work the authors report a common ubiquitin-mediated response to microsporidia and Orsay virus infection in *C*. *elegans*. Regarding F26F2.3, there is co-expression interaction with cluster V that represents co-expressed genes involved in stress response and metal toxicity^24^. Finally, F26F2.3 interacts physically with two genes from yeast, two hybrid studies (*lec-1* and C50F4.1) from the HUPO Protein Standard Initiative. *lec-1* is a protein binding to galactose^25^ while C50F4.1 has been shown to be part of an evolutionary-conserved set of intestinal genes that are important for feeding and response to pathogens^26^. Given the fact that Orsay virus infection leads to intestinal damage^12^ it is not surprising that this type of gene is connected to the gene list of unknown function under study. In addition, we found a text-mining interaction between B0507.8 and a set of genes representing cluster VI that were reported as a list of evolutionary-conserved genes involved in circadian rhythm regulation of olfaction in *C*. *elegans* linking Orsay virus infection with *C*. *elegans* behavior^27^. Since all of the 17 uncharacterized genes are closely co-expressed, it might be concluded that they are involved in a novel biological process that remains to be discovered. Among them, we identified 7 genes (B0507.8, B0507.10, CELE_T26F2.3, CELE_C17H1.6, CELE_C17H1.7, CELE_Y75B8A.39, and CELE_B0284.4) to be shared with other *Caenorhabditis* nematodes that might be attesting to their conserved specificity to this genus and/or reminiscent of an evolutionary conserved pathway.

Additionally, the 17 uncharacterized genes set were then analyzed for their enriched function using GSEA in PANTHER. Only a few of these genes were assigned to a known biological process such as kinases, the hormonally and chemically regulated *sdz* gene, and *cyp450* gene which are receptors known to be involved in toxicity pathways.

**Figure 2:**
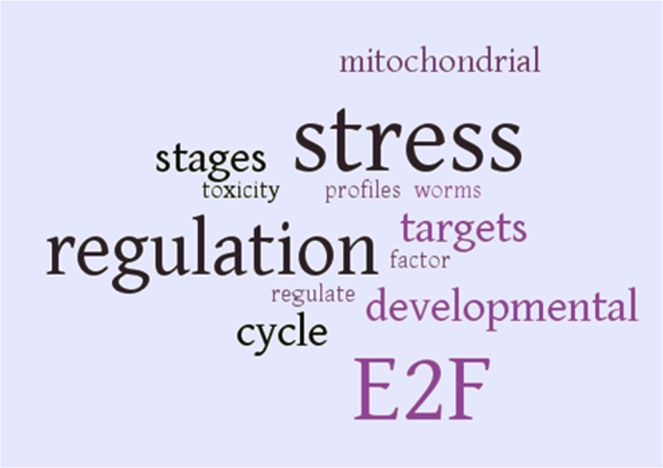
Word cloud analysis of all abstracts in GEO database referring to the 17 uncharacterized genes with a differential-expression filter on. Words were increased in size the more times they were mentioned in text. Stress, metal toxicity, development, and E2F transcription factor are seen to be enriched through this text mining approach. It should be noted that words that were important, but extensively repeated due to the subject of their papers, were removed. These words include: heme, HRG, LIN, cell, cadmium, pocket and transcription.

In addition, the GEO database^28^ was queried to identify studies in which the expression levels of the 17 uncharacterized genes were affected to gain insight into the context of these studies and obviously to better characterize the function of these genes. For this purpose, word cloud analysis of all the abstracts referring to the GEO datasets (n = 10) combined from each gene query (n = 17) was performed. Accordingly, differential expression of the 17 uncharacterized genes was present in studies related to stress, metal toxicity response and development, while the E2F transcription factor was enriched (Supplementary Table S1 and Fig. 2). This result is consistent with the finding from STRING reporting the putative involvement of these genes into similar biological processes and as result pathways. Moreover, out of 10 studies, one of them reporting metal toxicity for cadmium^24^ was the same study as the one identified by STRING for cluster V interacting with gene F26F2.3. Therefore, the co-expression of these genes and their possible targeting by the E2F transcription factor, suggests their involvement in a novel stress-related or immune response pathway. Thus, we propose that this set of co-expressed genes, act as putative markers for stress and immune response in *C*. *elegans*. Further functional studies are needed to unravel this pathway.

## Conclusion

*C*. *elegans* is one of the best model organisms for understanding the biology in all eukaryotes, including humans. It is also a powerful genetic tool to greatly accelerate future discoveries in human health. The establishment of a viral model system by Orsay virus in N2 Bristol strain opens unique prospects to identify novel pathway components of viral immunity. In this study, we showed that the 17 most differentially-expressed genes through transcriptomics analysis of datasets of viral infected *C*. *elegans* by Orsay virus, might be specific to a novel stress response. Through the use of structure prediction, we were able to obtain many accurate models that provided a framework to further determine the function of these genes. Most of the uncharacterized genes were folding as protein-α but one of these genes, T26F2.3 was found to be a Dom3Z Exoribonuclease (α/β- protein) homologue. STRING analysis and GEO annotation combined with Word cloud analysis provided further consensual insight that reinforced their possible involvement in stress, development and toxicity. The results provide a basis for additional experimental studies to unravel likely novel biological pathway components.

## Methods

### Transcriptomics analysis

**Figure 3:**
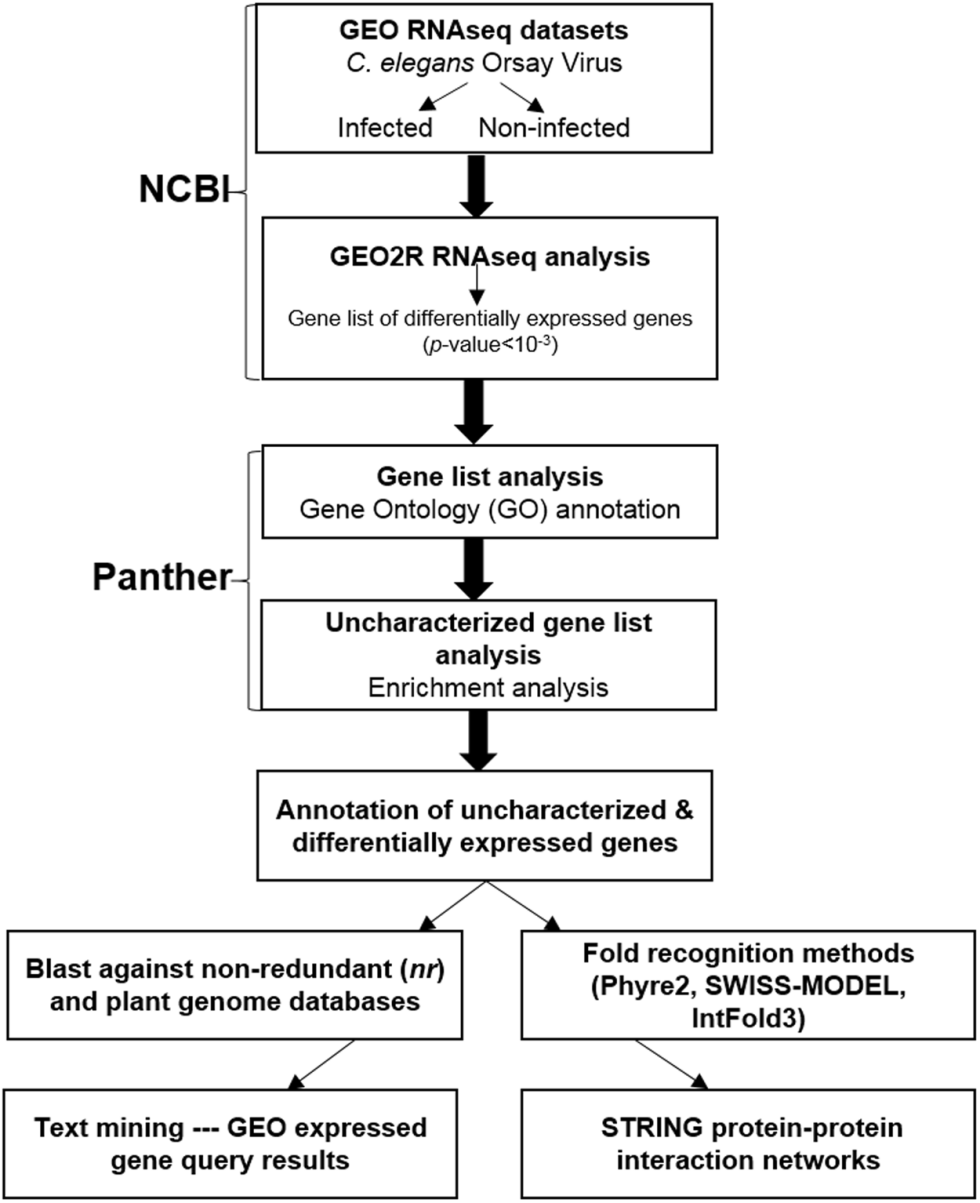
Pipeline of the meta-analysis approach.

In the pipeline presented in this study we used the GEO2R package suite at NCBI (Fig. 3). In short, GEO datasets from *C*. *elegans* infected by Orsay virus (GSE41056) were processed using the Bioconductor RNAseq analysis tools available in ‘LIMMA’^29^, ‘Biobase’^30^ and ‘GEOquery’^31^ packages implemented in R^32^. Differential-expression analysis was performed by assigning quadruplet RNAseq data sets to two different sample groups defined as infected and non-infected. The top 250 genes found to be the most significantly, differentially expressed were then ranked from their lowest to highest *p*-value. Using this approach, the 250 differentially expressed genes identified were within an adjusted *p*-value ranging from 9.82e-11 to 9.88e-04. The *p*-values were adjusted by Bonferroni correction for multiple testing according to the method proposed by Benjamini and colleagues^33^.

### GO annotation

Gene Set Enrichment Analysis (GSEA) was carried out using PANTHER^15^ to gather insights into the function and the biological pathway of the differentially-expressed genes. From a provided gene list their annotation using Gene Ontology (GO) is determined as well as the over representation of the GO terms is evaluated by calculating an enrichment score. This parameter determines if a giving gene list is enriched in a particular Gene Function, Biological Process or Cellular Localization relative to a control (https://www.pantherdb.org/).

### 3D-model building by comparative modeling

Fold recognition method PHYRE2 was used to assign the functions to the 17 uncharacterized genes based on the 3D-structural model calculated by comparative modeling. In complement, other fold recognition methods were used when fold prediction failed by PHYRE2^17^ such as SWISS-MODEL^18^ and the IntFOLD3^19^ servers. To assess the quality of the modeling, an unbiased approach that allowed direct comparison across the different methods was used. The Root-Mean-Square Deviation (RMSD) of atomic positions using spdbv^34^ (https://spdbv.vital-it.ch/) were determined between the atomic coordinates of the modeled and the template structures. Because, the RMSD value can be very low when only few equivalent Cα atoms are being superimposed, we also considered how much of the sequence of unknown structure is being modeled as determined by the percentage coverage. Once a good structural identity using these two parameters was obtained between the model and the template, functional annotation transfer (from the template to the model) was considered to get insights into the function of these uncharacterized protein sequences.

### STRING Protein-protein interactions

The STRING (Search Tool for the Retrieval of Interacting Genes/Proteins) database^35^ (https://string-db.org) was then used to construct protein-protein interaction networks between all the 17 genes to explore further their function. A network representation of the first shell of interactions capturing seven types of evidence was visualized in STRING. Our setting included maximizing the network representation to the first shell of interactors. As a result, all the possible partners of the query proteins listed in the database were added. The lower bound threshold for the minimal interactions score was set to a cutoff of 0.4 determining the inclusion/exclusion limit for an interactor to be considered and added to the network.

### GEOexpress queries

For the purpose of GEOexpress^28^ queries analysis, each of the 17 gene names was used as keyword to query and identify which GEO datasets had their gene expression changed by filtering the query for up or down regulation^28^. For the genes that came up with “no results found” the procedure was repeated without the filtering step. This method could check whether the gene was constitutively expressed, or whether the gene simply did not exist within the data set. All the retrieved abstracts of the GEO dataset through the 17 individual searches were pooled and later subjected to a text mining word cloud approach.

## Supplementary information

Supporting Information

## Data Availability

All the data-sets used in the study are available to the users on request through the corresponding author (F. Pio).
