# Supplementary material for: Meta-analysis suggests evidence of novel stress-related pathway components in Orsay virus - Caenorhabditis elegans viral model
Source: Sci Rep. 2019 Mar 13;9:4399. doi: 10.1038/s41598-019-40762-9 (PMC6416287; doi:10.1038/s41598-019-40762-9)
Supplement: Supplementary file 1 — Supporting Information [file 41598_2019_40762_MOESM1_ESM.doc]

**Supporting Information**

**Meta-analysis suggests evidence of novel stress-related pathway components in Orsay virus – *Caenorhabditis elegans* viral model**

**Priyanka Mishra^1^, Jessica Ngo^1^, Jahanshah Ashkani^1^ & Frederic Pio^1*^**

^1^ Molecular Biology and Biochemistry Department, Simon Fraser University,

Burnaby-V5A1S6, British Columbia, Canada

**Table of Contents**

1. **Supplementary Figure S1** Predicted structures obtained using PHYRE2, of the 17 unknown and highly differentially expressed genes. Below the Gene-Template pdb code, the RMSD value is displayed as computed by superimposing the best template with the model. (): Percent coverage of the *C. elegans* protein sequence by the model. **Page 2-3**
2. **Supplementary Figure S2** Predicted structures obtained using SWISS-MODEL, of the 17 unknown and highly differentially expressed genes. Below the Gene-Template pdb code, the RMSD value is displayed as computed by superimposing the best template with the model. (): Percent coverage of the *C. elegans* protein sequence by the model. N.T.: No template found. **Page 4-5**
3. **Supplementary Figure S3** Predicted structures obtained using IntFOLD3, of the 17 unknown and highly differentially expressed genes. Below the Gene-Template pdb code, the RMSD value is displayed as computed by superimposing the best template with the model. (): Percent coverage of the *C. elegans* protein sequence by the model. **Page 6-7**
4. **Supplementary Figure S4** Predicted structures obtained from Phyre2 (pink), SWISS-MODEL (green), and IntFOLD3 (blue) servers that did not converge into a single secondary structure or fold across the three programs. **Page 8**
5. **Supplementary Figure S5** Predicted structures from Phyre25 (pink), SWISS-MODEL (green), and IntFOLD3 (blue) servers that converged into a single secondary structure (alpha-helix), but not a single fold across the three programs. **Page 9**
6. **Supplementary Figure S6** Predicted structures from Phyre2 (pink), SWISS-MODEL (green), and IntFOLD3 (blue) servers that converged into a single fold across the three programs. This fold was obtained using the Dom3Z mouse protein exonuclease as template (3fqi) for both Phyre and INTFOLD3. While this template was also a high match for the SWISS-MODEL server, the protein that was used as the template, Rai1 from *Scheffersomyces stipitis* has a slightly higher sequence similarity, and so was used instead. This alternative template still has the same fold but functions as a hydrolase instead that can adopt a variety of folding patterns. **Page 10**
7. **Supplementary Table S1** Results for the 17 uncharacterized genes upon querying the genes in the GEO dataset and filtering for up/down regulated genes. As a result genes involved in any possible housekeeping functions were filtered. Note: v.d. means that gene expression varies during development.  **Page 11**

| **B0507.8 – 2fxm**  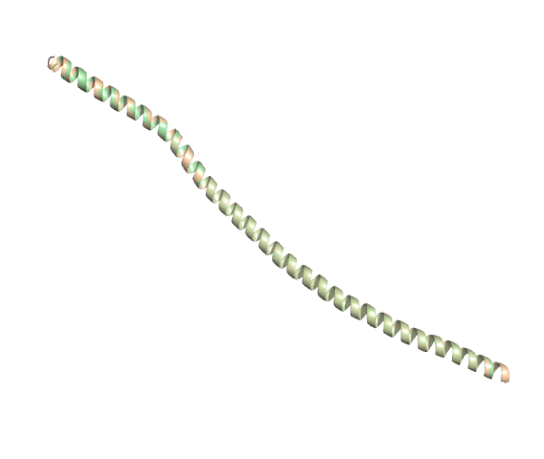**0 (26)** | **F26F2.4 isoform a – 2w3y**  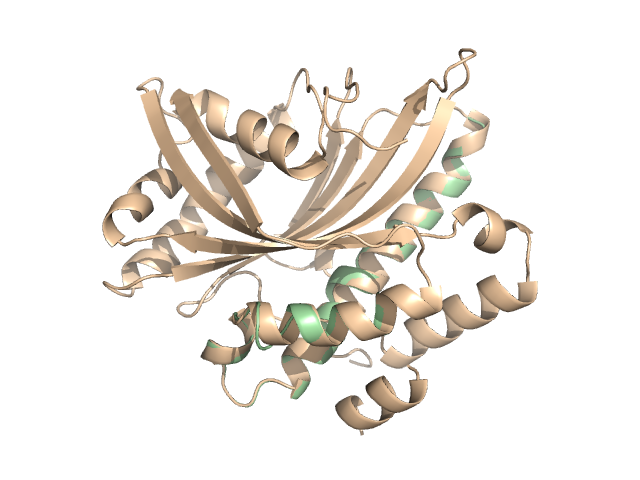**0.55 (66)** | **F26F2.4 isoform b – 2w3y**  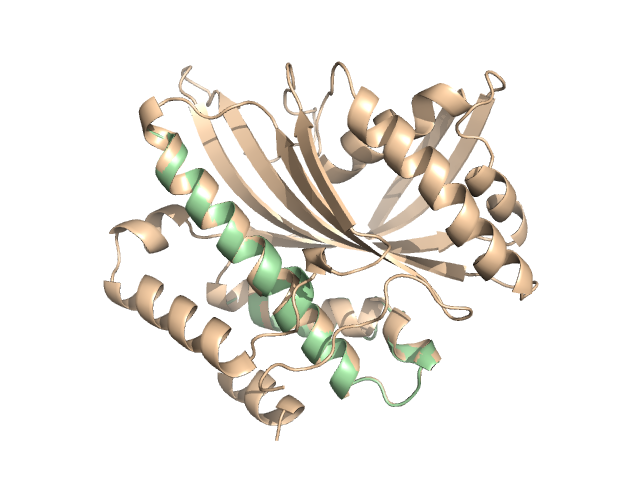**0.55 (55**) |
| --- | --- | --- |
| **F26F2.5 – 3pf6**  **0.67 (14)**  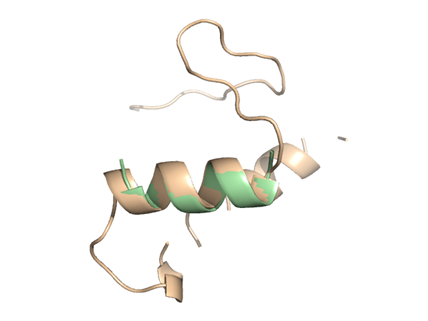 | **B0507.10 – 1deq**  **0.03 (26)**  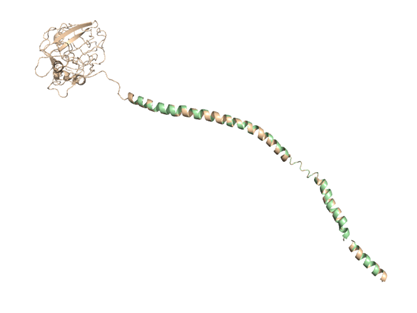 | **CELE_T26F2.3 – 3fqi**  **0.28 (99)**  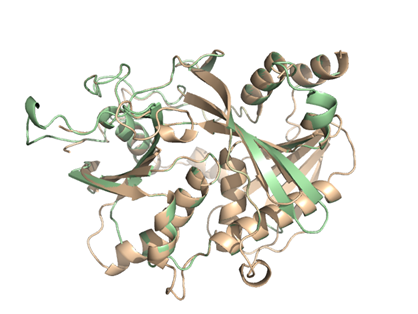 |
| **CELE_C43D7.4 – 1zru**  **0.51 (21)**  **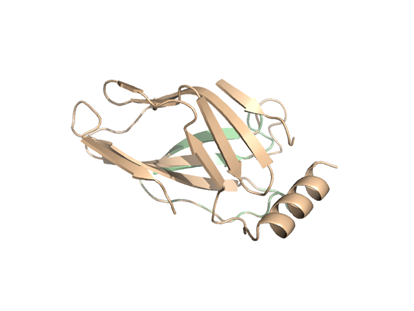** | **CELE_C17H1.6 – 3ghg**  **1.33 (36)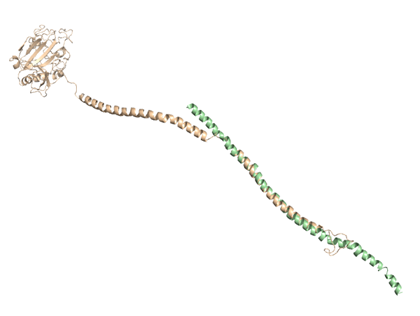** | **CELE_C17H1.7 – 3ghg**  **0 (26)**  **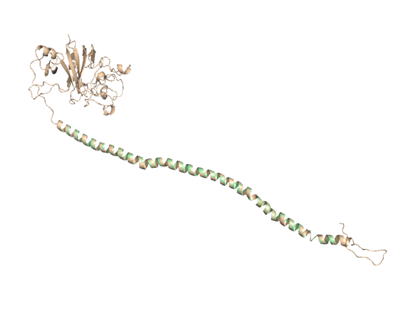** |
|  | **CELE_Y75B8A – 3a5t**  **0 (22)**  **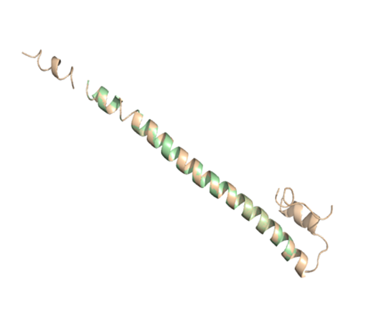** |  |

| **F26F2.2 – 1x5w**  **0 (17)**  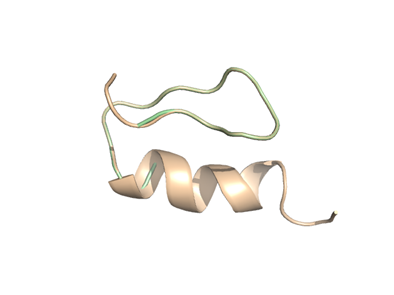 | **CELE_C43D7.7 – 4n3z**  **0 (17)**  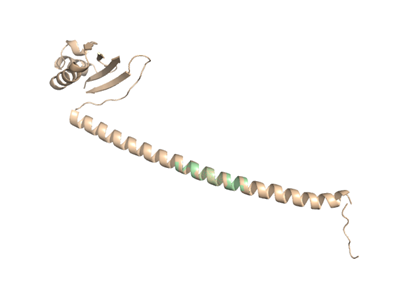 | **F26F2.3 – 3n9t**  **0.44(18)**  **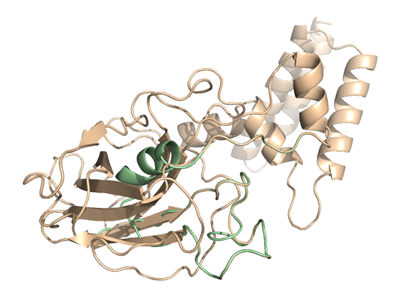** |
| --- | --- | --- |
| **F26F2.1 – 4zrk**  **0.02 (1)**  **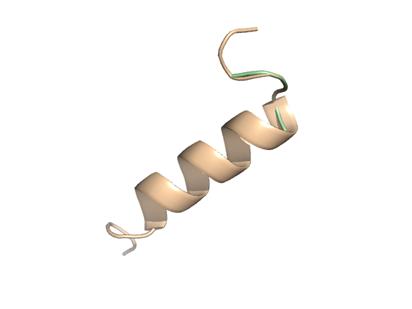** | **C49C8.2 – 1etf**  **0 (5)**  **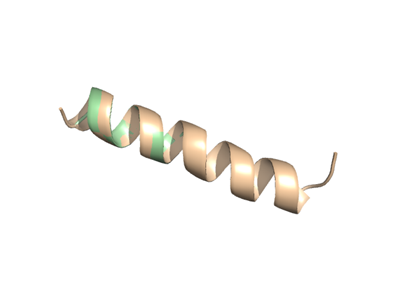** | **CELE_B0284.4 – 2l81**  **0 (31)**  **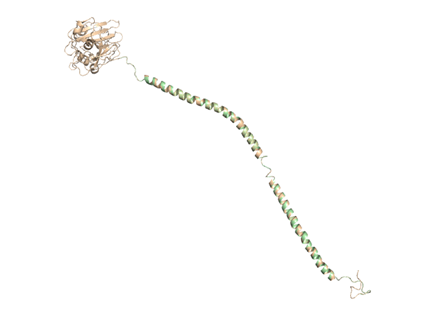** |
| **F42C5.3 isoform a – 1deq**  **0.03 (35)**  **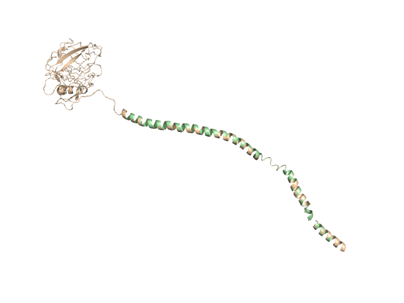** | **F42C5.3 isoform b – 2jv7**  **0 (4)**  **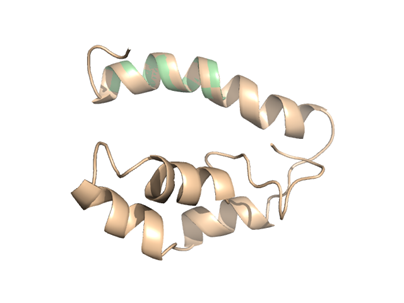** | **Sdz-6 – 3j3a**  **0 (14)**  **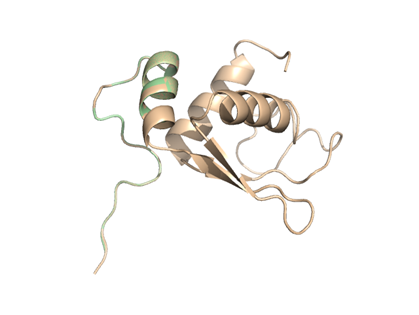** |

**Supplementary Figure S1** Predicted structures obtained using PHYRE2, of the 17 unknown and highly differentially expressed genes. Below the Gene-Template pdb code, the RMSD value is displayed as computed by superimposing the best template with the model. (): Percent coverage of the *C. elegans* protein sequence by the model.

| **B0507.8 – 3ck6**  **0.45 (25)**  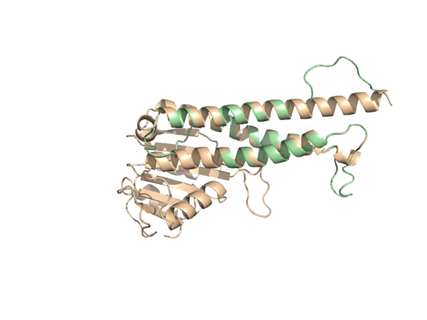 | **F26F2.4 isoform a – 4g78**  **0.5 (32)**  **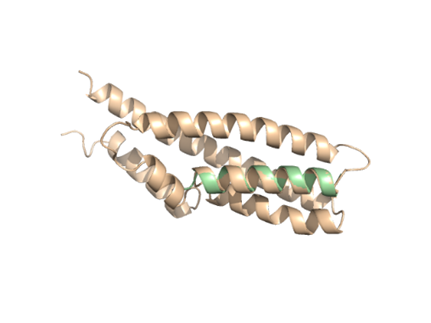** | **F26F2.4 isoform b – N.T.** |
| --- | --- | --- |
| **F26F2.5 – 4g78**  **0.53 (32)**  **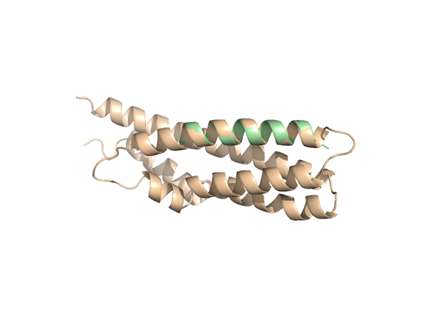** | **B0507.10 – 5fm1**  **0.26 (32)**  **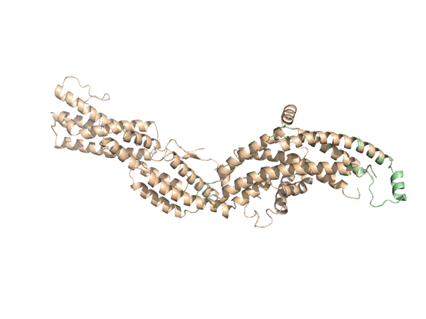** | **CELE_T26F2.3 – 5bto**  **0.44 (96)**  **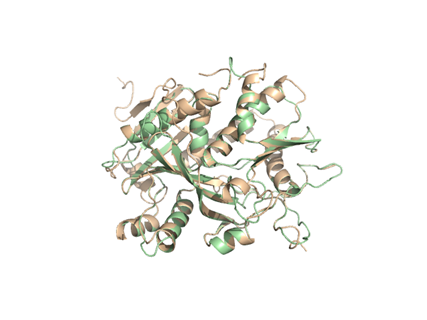** |
| **CELE_C43D7.4 – 4j3d**  **0.28 (25)**  **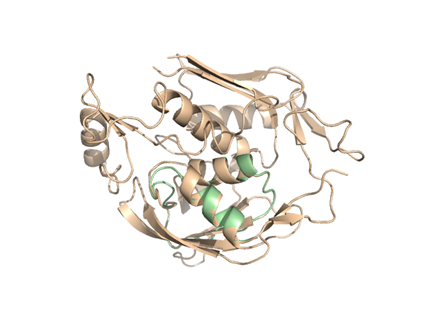** | **CELE_C17H1.6 – 4ilo**  **0.1 (31)**  **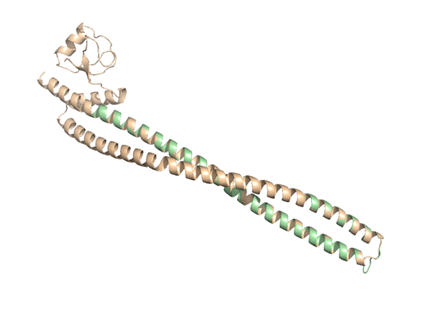** | **CELE_C17H1.7 – 1i84**  **0.38 (73)**  **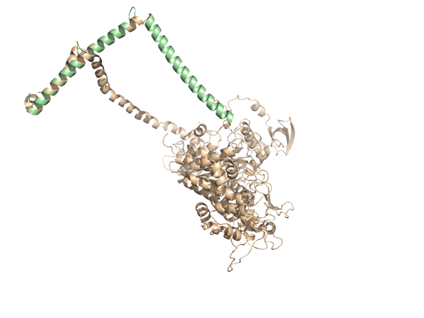** |
|  | **CELE_Y75B8A – 2e42**  **0.06 (23)**  **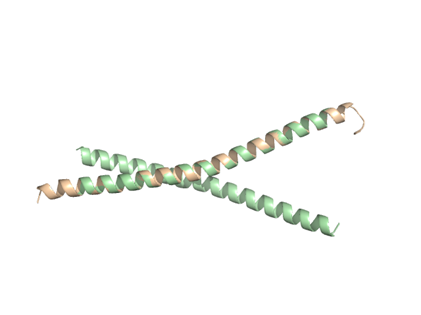** |  |

| **F26F2.2 – 2k5c**  **1.42 (46)**  **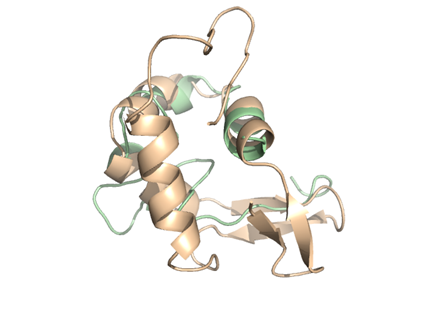** | **CELE_C43D7.7 – 4n3z**  **0.09(59)**  **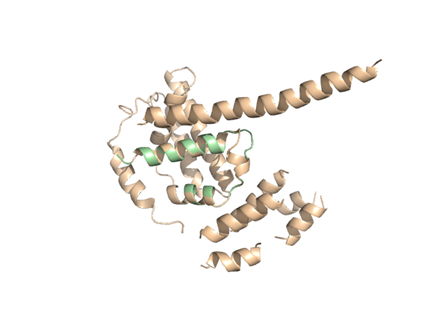** | **F26F2.3 – 4cxi**  **0.31 (15)**  **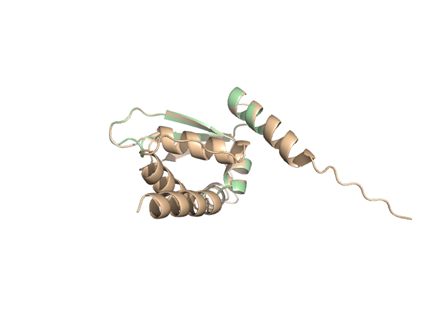** |
| --- | --- | --- |
| **F26F2.1 – 4cih**  **0.18 (6)**  **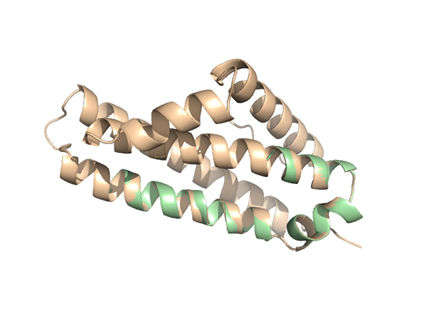** | **C49C8.2 – 2o7a**  **0.35 (35)**  **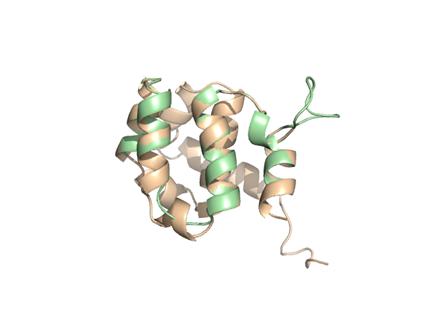** | **CELE_B0284.4 – 2l81**  **0.53 (28)**  **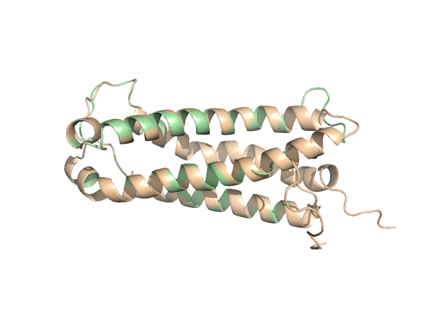** |
| **F42C5.3 isoform a – 4b2f**  **0.09 (17)**  **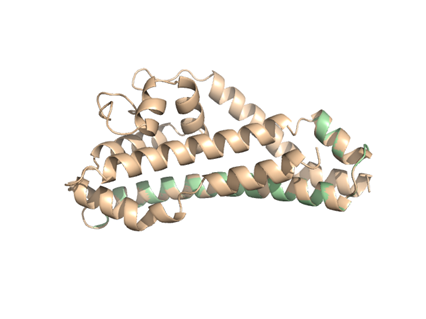** | **F42C5.3 isoform b – 4wik**  **0.21 (22)**  **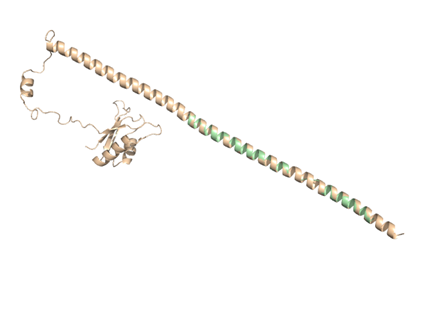** | **Sdz-6 – 2yy8**  **0.07 (26)**  **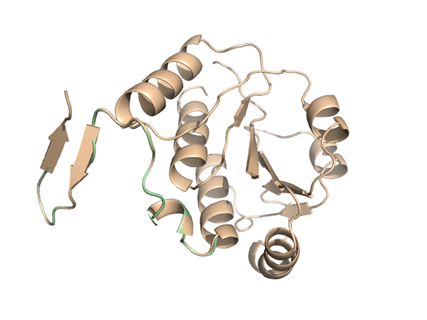** |

**Supplementary Figure S2** Predicted structures obtained using SWISS-MODEL, of the 17 unknown and highly differentially expressed genes. Below the Gene-Template pdb code, the RMSD value is displayed as computed by superimposing the best template with the model. (): Percent coverage of the *C. elegans* protein sequence by the model.. N.T.: No template found.

| **B0507.8 – 4c0o**  **1.3 (98)**  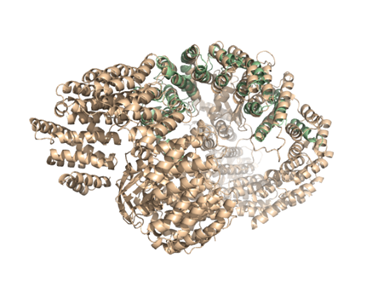 | **F26F2.4 isoform a – 5hda**  **1.38 (82)**  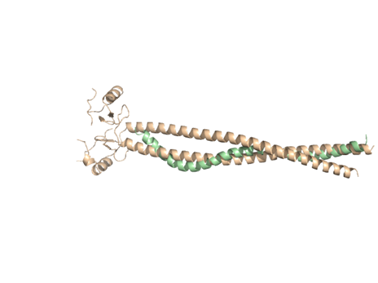 | **F26F2.4 isoform b – 4yto**  **4.78 (90)**  **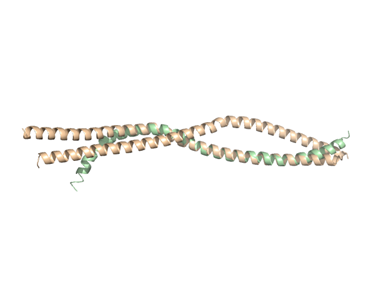** |
| --- | --- | --- |
| **F26F2.5 – 5hda**  **4.04 (96)**  **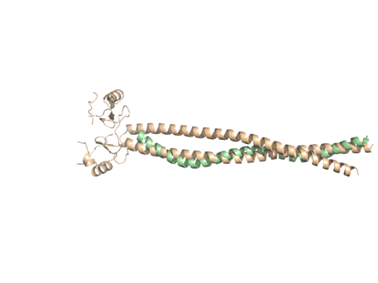** | **B0507.10 – 3edv**  **1.73 (80)**  **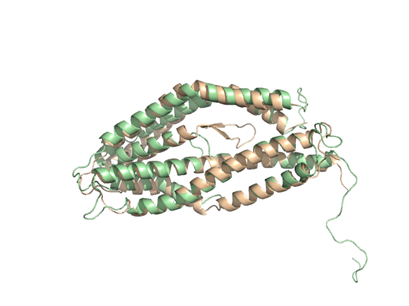** | **CELE_T26F2.3 – 3fqj**  **1.02 (97)**  **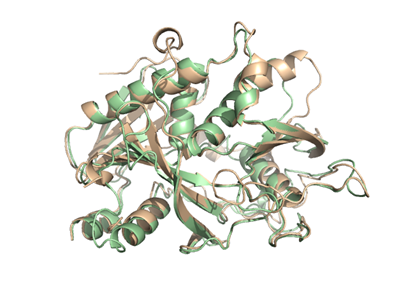** |
| **CELE_C43D7.4 – 2il6**  **0.65 (90)**  **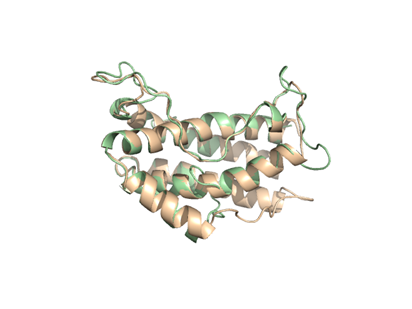** | **CELE_C17H1.6 – 4u0q**  **1.14 (87)**  **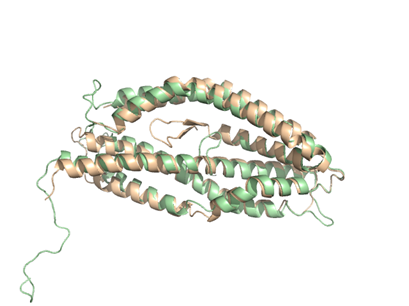** | **CELE_C17H1.7 – 5cwm**  **0.52 (55)**  **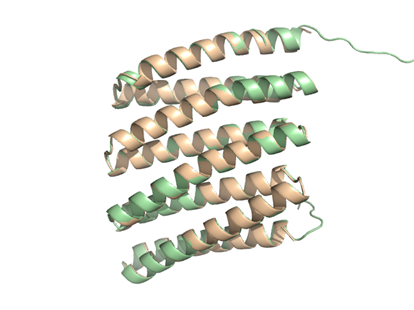** |
|  | **CELE_Y75B8A – 4tql**  **1.28 (100)**  **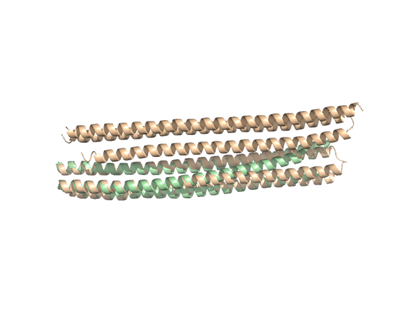** |  |
| **F26F2.2 – 2qih**  **5.22 (87)**  **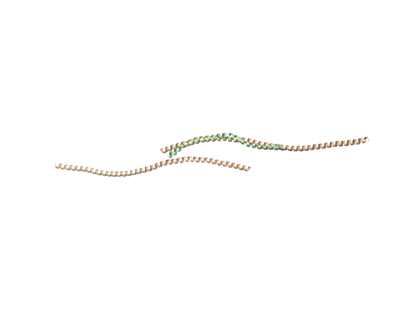** | **CELE_C43D7.7 – 3vwb**  **1.28 (93)**  **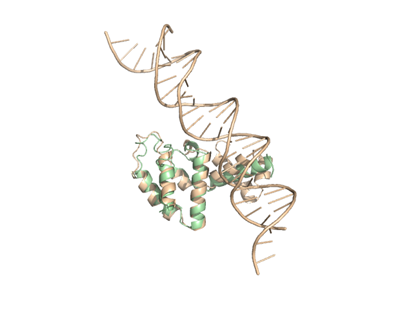** | **F26F2.3 – 4yxz**  **0.81 (95)**  **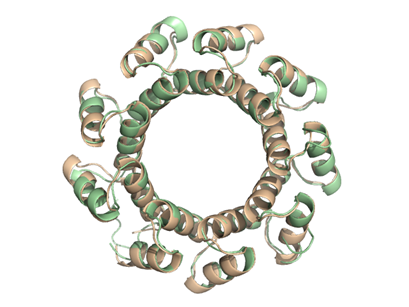** |
| **F26F2.1 – 2ocw**  **N.A. (0)**  **No template model – only alpha carbon trace** | **C49C8.2 – 1ls4**  **1.75 (90)**  **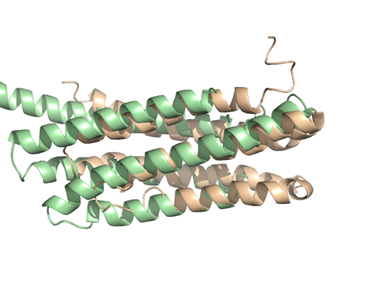** | **CELE_B0284.4 – 4uxv**  **1.45 (90)**  **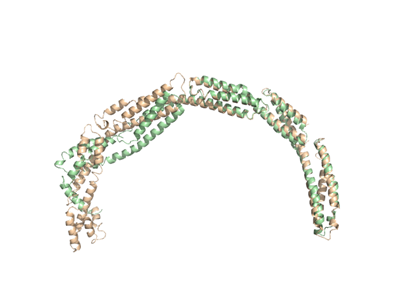** |
| **F42C5.3 isoform a – 4u0q**  **0.87 (91)**  **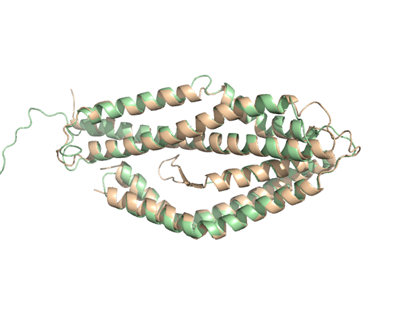** | **F42C5.3 isoform b – 4wpe**  **1.07 (86)**  **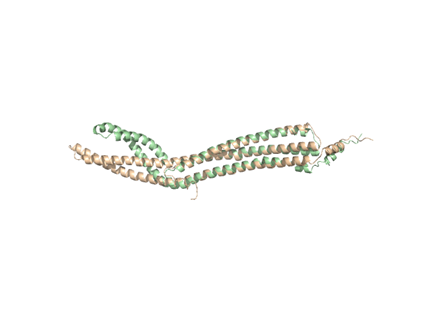** | **Sdz-6 – 4oje**  **0.62 (82)**  **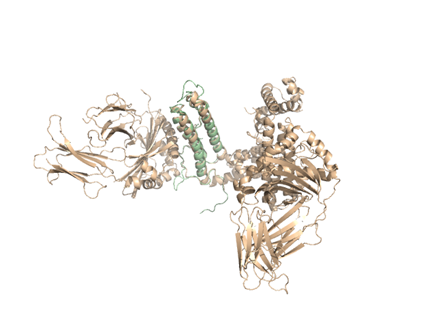** |

**Supplementary Figure S3** Predicted structures obtained using IntFOLD3, of the 17 unknown and highly differentially expressed genes. Below the Gene-Template pdb code, the RMSD value is displayed as computed by superimposing the best template with the model. (): Percent coverage of the *C. elegans* protein sequence by the model.

| **CELE_C43D7.4 1zru, 4j3d, 2il6** | **F26F2.1*  4cih, 4zrk, 2ocw** | **F26F2.2* 1x5w, 2k5c, 2qih** |
| --- | --- | --- |
| 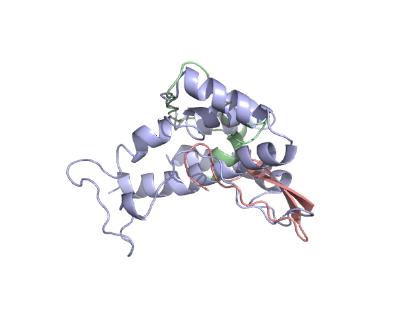 | 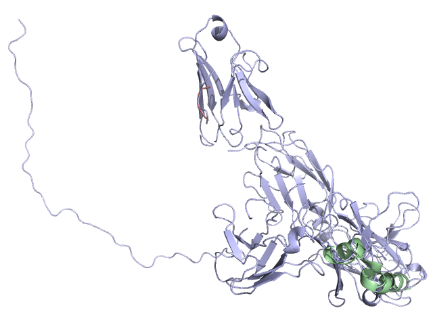 | 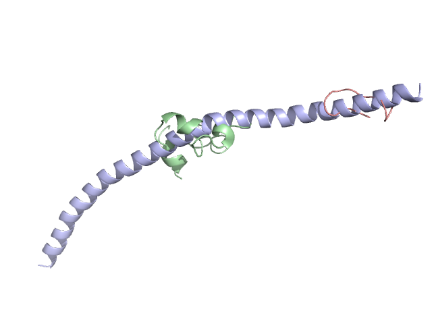 |

*The Phyre model is only a small chain, and may be hard to distinguish here.

**Supplementary Figure S4** Predicted structures obtained from Phyre2 (pink), SWISS-MODEL (green), and IntFOLD3 (blue) servers that did not converge into a single secondary structure or fold across the three programs.

| **B0507.8 2fxm, 3ck6, 4c0o** | **B0507.10 1deq, 5fm1, 3edv** | **C49C8.2 1etf, 2o7a, 1ls4** | **CELE_B0284.4 3ghg, 2l81, 4uxv** |
| --- | --- | --- | --- |
| **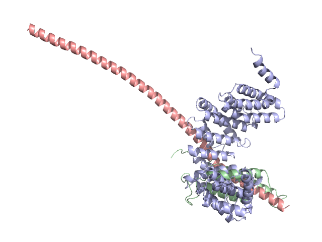** | 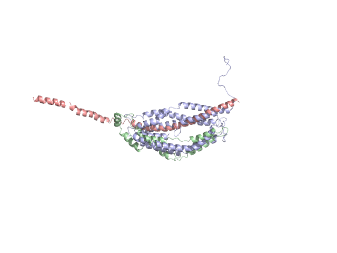 | 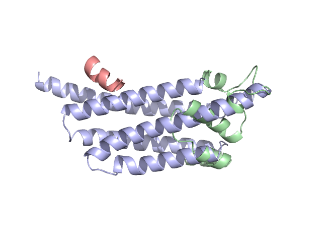 | 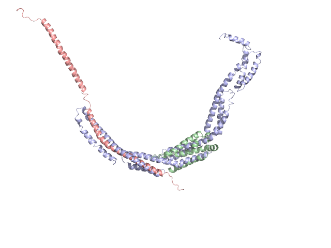 |
| **C17H1.6 3ghg, 4ilo, 4u0q** | **C17H1.7 3ghg, 1i84, 5cwm** | **C43D7.7 3n4x, 4n3z, 4vw** | **CELE_Y75B8A.39 3a5t, 2e42** |
| 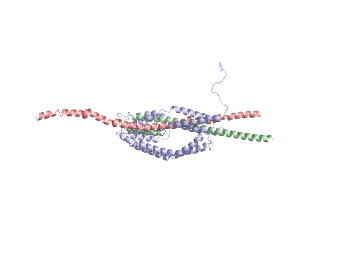 | 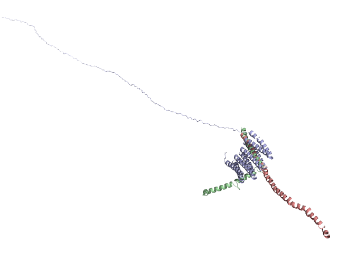 | 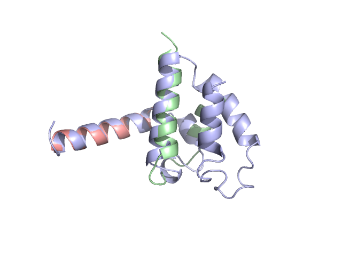 | 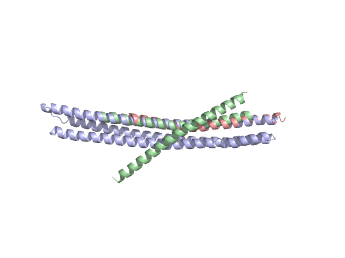 |
| **F26F2.3 3n9t, 4cx, 4yxz** | **F26F2.4 isoform a 2w3y, 4g78, 5hda** | **F26F2.4 isoform b* 2w3y, –, 4yto** | **F26F2.5 3pf6, 4g78, 5hda** |
| 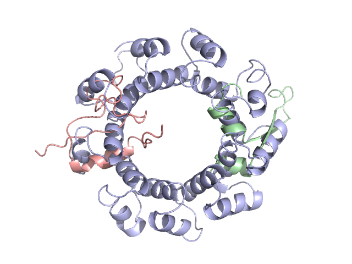 | 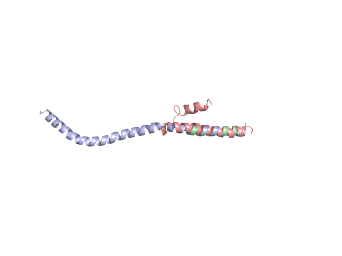 | 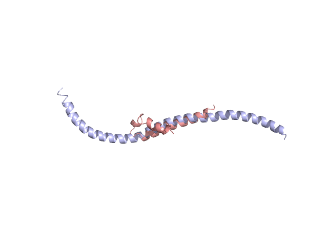 | 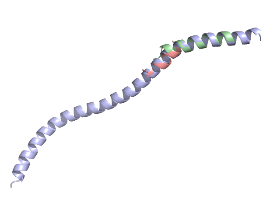 |
| **F42C5.3 isoform a 1deq, 4b2f, 4u0q** | **F42C5.3 isoform b 2jv7, 4wik, 4wpe** | **sdz-6 3j3a, 2yy8, 4oje** |  |
| 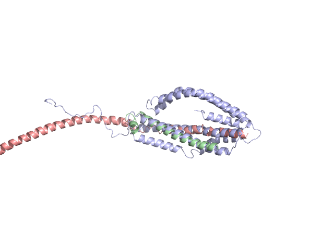 | 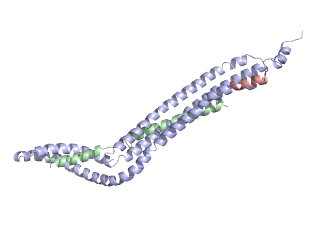 | 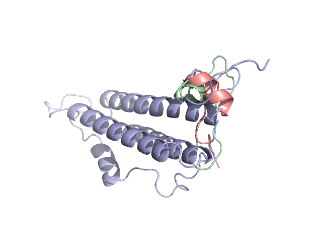 |  |

*No suitable template for the gene was found in the SWISS-MODEL server.

**Supplementary Figure S5** Predicted structures from Phyre25 (pink), SWISS-MODEL (green), and IntFOLD3 (blue) servers that converged into a single secondary structure (alpha-helix), but not a single fold across the three programs.

| **CELE_T26F2.3 3fqi, 5bto, 3fqi** |
| --- |
| 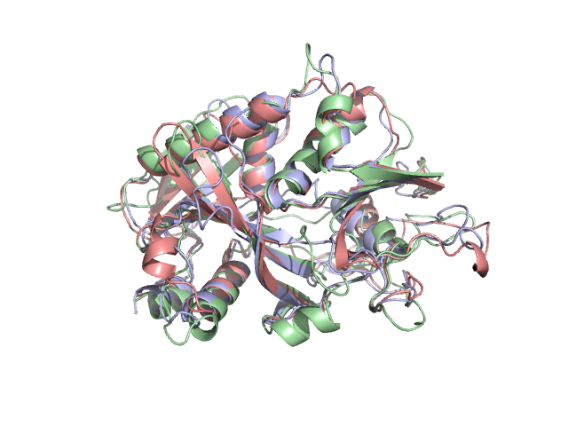 |

**Supplementary Figure S6** Predicted structures from Phyre2 (pink), SWISS-MODEL (green), and IntFOLD3 (blue) servers that converged into a single fold across the three programs. This fold was obtained using the Dom3Z mouse protein exonuclease as template (3fqi) for both Phyre and INTFOLD3. While this template was also a high match for the SWISS-MODEL server, the protein that was used as the template, Rai1 from *Scheffersomyces stipitis* has a slightly higher sequence similarity, and so was used instead. This alternative template still has the same fold but functions as a hydrolase instead that can adopt a variety of folding patterns.

| **Gene** | **Accession**  **number** | **Dataset** | **Up/down**  **regulated** | **Gene description** |
| --- | --- | --- | --- | --- |
| B0507.8 | [GSE7535](http://www.ncbi.nlm.nih.gov/geo/query/acc.cgi?acc=GSE7535) | [Cadmium exposure: time course](http://www.ncbi.nlm.nih.gov/geoprofiles/56453616) | Up | Toxicity & stress response, and development |
|  | [GSE42192](http://www.ncbi.nlm.nih.gov/geo/query/acc.cgi?acc=GSE42192) | [Dietary probiotic Lactobacillus effect on N2 wildtype strain: adult developmental stage](http://www.ncbi.nlm.nih.gov/geoprofiles/109443296) | v.d.* |  |
|  | [GSE34471](http://www.ncbi.nlm.nih.gov/geo/query/acc.cgi?acc=GSE34471) | [Heme-responsive gene-2 deficient mutant](http://www.ncbi.nlm.nih.gov/geoprofiles/99332696) | Down |  |
|  | [GSE6547](http://www.ncbi.nlm.nih.gov/geo/query/acc.cgi?acc=GSE6547) | [Pocket protein ortholog lin-35 null mutant at various stages of development](http://www.ncbi.nlm.nih.gov/geoprofiles/39754496) | Up |  |
| F26F2.4  (isoform a & b) | [GSE6547](http://www.ncbi.nlm.nih.gov/geo/query/acc.cgi?acc=GSE6547) | [Pocket protein ortholog lin-35 null mutant at various stages of development](http://www.ncbi.nlm.nih.gov/geoprofiles/39752545) | v.d.* | Development |
| F26F2.5 | No results | | | |
| B0507.10 | [GSE7535](http://www.ncbi.nlm.nih.gov/geo/query/acc.cgi?acc=GSE7535) | [Cadmium exposure: time course](http://www.ncbi.nlm.nih.gov/geoprofiles/56452733) | Up | Toxicity response & development, |
|  | [GSE34471](http://www.ncbi.nlm.nih.gov/geo/query/acc.cgi?acc=GSE34471) | [Heme-responsive gene-2 deficient mutant](http://www.ncbi.nlm.nih.gov/geoprofiles/99339050) | Down |  |
|  | [GSE2862](http://www.ncbi.nlm.nih.gov/geo/query/acc.cgi?acc=GSE2862) | [Basic helix-loop-helix transcription factor Twist overexpression](http://www.ncbi.nlm.nih.gov/geoprofiles/33157050) | Down |  |
|  | [GSE6547](http://www.ncbi.nlm.nih.gov/geo/query/acc.cgi?acc=GSE6547) | [Pocket protein ortholog lin-35 null mutant at various stages of development](http://www.ncbi.nlm.nih.gov/geoprofiles/39760850) | Up |  |
| CELE_T26F2.3 | [GSE21784](http://www.ncbi.nlm.nih.gov/geo/query/acc.cgi?acc=GSE21784) | [Effect of aging on nematode](http://www.ncbi.nlm.nih.gov/geoprofiles/73559682) | Up | Stress response & development |
|  | [GSE38196](http://www.ncbi.nlm.nih.gov/geo/query/acc.cgi?acc=GSE38196) | [Mitochondrial stress effect on ATFS-1 mutants](http://www.ncbi.nlm.nih.gov/geoprofiles/99487382) | Up |  |
| CELE_C43D7.4 | No results | | | |
| CELE_C17H1.6 | No results | | | |
| CELE_C17H1.7 | [GSE1762](http://www.ncbi.nlm.nih.gov/geo/query/acc.cgi?acc=GSE1762) | [Long-lived daf-2 insulin/IGF-1 receptor mutant expression profiling](http://www.ncbi.nlm.nih.gov/geoprofiles/6270416) | Down | Stress response & development |
|  | [GSE34471](http://www.ncbi.nlm.nih.gov/geo/query/acc.cgi?acc=GSE34471) | [Heme-responsive gene-2 deficient mutant](http://www.ncbi.nlm.nih.gov/geoprofiles/99336916) | Down |  |
|  |  | [Mitochondrial stress effect on ATFS-1 mutants](http://www.ncbi.nlm.nih.gov/geoprofiles/99486516) | Up |  |
|  | [GSE2862](http://www.ncbi.nlm.nih.gov/geo/query/acc.cgi?acc=GSE2862) | [Basic helix-loop-helix transcription factor Twist overexpression](http://www.ncbi.nlm.nih.gov/geoprofiles/33154916) | Down |  |
|  | [GSE6547](http://www.ncbi.nlm.nih.gov/geo/query/acc.cgi?acc=GSE6547) | [Pocket protein ortholog lin-35 null mutant at various stages of development](http://www.ncbi.nlm.nih.gov/geoprofiles/39758716) | Up |  |
| CELE_Y75B8A.39 | [GSE42192](http://www.ncbi.nlm.nih.gov/geo/query/acc.cgi?acc=GSE42192) | [Dietary probiotic Lactobacillus effect on N2 wildtype strain: adult developmental stage](http://www.ncbi.nlm.nih.gov/geoprofiles/109454750) | v.d.* | Stress response & development |
|  | [GSE38196](http://www.ncbi.nlm.nih.gov/geo/query/acc.cgi?acc=GSE38196) | [Mitochondrial stress effect on ATFS-1 mutants](http://www.ncbi.nlm.nih.gov/geoprofiles/99493750) | Up |  |
| F26F2.2 | No results | | | |
| CELE_C43D7.7 | No results | | | |
| F26F2.3 | [GSE6547](http://www.ncbi.nlm.nih.gov/geo/query/acc.cgi?acc=GSE6547) | [Pocket protein ortholog lin-35 null mutant at various stages of development](http://www.ncbi.nlm.nih.gov/geoprofiles/39759665) | v.d. * | Development |
| F26F2.1 | No results | | | |
| C49C8.2 | [GSE8159](http://www.ncbi.nlm.nih.gov/geo/query/acc.cgi?acc=GSE8159) | [Embryonic motor neurons](http://www.ncbi.nlm.nih.gov/geoprofiles/40752584) | Up | Embryonic development |
| CELE_B0284.4 | No results | | | |
| F42C5.3  (isoform a & b) | GSE7535 | Cadmium exposure: time course | Up | Toxicity & stress response, and development |
| sdz-6 | [GSE1762](http://www.ncbi.nlm.nih.gov/geo/query/acc.cgi?acc=GSE1762) | [Long-lived daf-2 insulin/IGF-1 receptor mutant expression profiling](http://www.ncbi.nlm.nih.gov/geoprofiles/6269799) | Up | Development |

**Supplementary Table S1 Results for the 17 uncharacterized genes upon querying the genes in the GEO dataset and filtering for up/down regulated genes. As a result genes involved in any possible housekeeping functions were filtered. Note: v.d. means that gene expression varies during development.**
